# 2-(4-Methoxy­phen­yl)-6-trifluoro­methyl-1*H*-pyrrolo[3,2-*c*]quinoline monohydrate

**DOI:** 10.1107/S1600536810013644

**Published:** 2010-04-17

**Authors:** Grzegorz Dutkiewicz, Anil N. Mayekar, H. S. Yathirajan, B. Narayana, Maciej Kubicki

**Affiliations:** a Department of Chemistry, Adam Mickiewicz University, Grunwaldzka 6, 60-780 Poznań, Poland; b Department of Studies in Chemistry, University of Mysore, Manasagangotri, Mysore 570 006, India; c Department of Studies in Chemistry, Mangalore University, Mangalagangotri 574 199, India

## Abstract

In the title compound, C_19_H_13_F_3_N_2_O·H_2_O, the phenyl and pyrroloquinoline ring system are close to coplanar [dihedral angle = 10.94 (4)°]. The meth­oxy group also is almost coplanar with the phenyl ring [5.4 (1)°]. In the crystal structure N—H⋯O(water) and water–quinoline O—H⋯N hydrogen bonds build up a supra­molecular chain-like arrangement along [001]. The remaining H atom of the water mol­ecule does not take part in classical hydrogen bonds. Instead, this O—H bond points toward the center of the phenyl ring of a neighbouring mol­ecule. Weak C—H⋯O and C—H⋯π inter­actions are also present.

## Related literature

For a description of the Cambridge Structural Database, see: Allen (2002 ▶). For O—H⋯π bonds, see: Atwood *et al.* (1991 ▶). For the graph-set description of hydrogen-bond systems, see: Bernstein *et al.* (1995 ▶). For the influence of substituents on the geometry of aromatic rings, see: Domenicano (1988 ▶). For a similar synthesis, see: Dutkiewicz *et al.* (2010 ▶). For related structures, see: Fan & Chen (1987 ▶); Lynch *et al.* (2001 ▶); Lynch & McClenaghan (2002 ▶). 
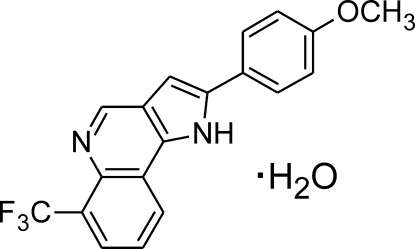


## Experimental

### 

#### Crystal data

C_19_H_13_F_3_N_2_O·H_2_O
*M* *_r_* = 360.33
Monoclinic, 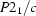

*a* = 13.838 (1) Å
*b* = 7.0432 (5) Å
*c* = 17.758 (2) Å
β = 102.743 (8)°
*V* = 1688.2 (2) Å^3^
*Z* = 4
Cu *K*α radiation
μ = 0.99 mm^−1^
*T* = 295 K
0.4 × 0.2 × 0.1 mm

#### Data collection

Oxford Diffraction SuperNova (single source at offset) Atlas diffractometer
Absorption correction: multi-scan (*CrysAlis PRO*; Oxford Diffraction, 2009 ▶) *T*_min_ = 0.340, *T*_max_ = 1.000
5601 measured reflections
3304 independent reflections
2767 reflections with *I* > 2σ(*I*)
*R*_int_ = 0.013

#### Refinement

*R*[*F* ^2^ > 2σ(*F* ^2^)] = 0.041
*wR*(*F* ^2^) = 0.128
*S* = 1.07
3304 reflections
296 parameters
All H-atom parameters refined
Δρ_max_ = 0.19 e Å^−3^
Δρ_min_ = −0.21 e Å^−3^

### 

Data collection: *CrysAlis PRO* (Oxford Diffraction, 2009 ▶); cell refinement: *CrysAlis PRO*; data reduction: *CrysAlis PRO*; program(s) used to solve structure: *SIR92* (Altomare *et al.*, 1993 ▶); program(s) used to refine structure: *SHELXL97* (Sheldrick, 2008 ▶); molecular graphics: *Stereochemical Workstation Operation Manual* (Siemens, 1989 ▶) and *Mercury* (Macrae *et al.*, 2008 ▶); software used to prepare material for publication: *SHELXL97*.

## Supplementary Material

Crystal structure: contains datablocks I, global. DOI: 10.1107/S1600536810013644/im2190sup1.cif

Structure factors: contains datablocks I. DOI: 10.1107/S1600536810013644/im2190Isup2.hkl

Additional supplementary materials:  crystallographic information; 3D view; checkCIF report

## Figures and Tables

**Table 1:** Hydrogen-bond geometry (Å, °) *CgA*, *CgB*, *CgD* are the centroids of the C5–C9,C1C, N1,C2–C5,C1C and C14–C19 rings, respectively.

*D*—H⋯*A*	*D*—H	H⋯*A*	*D*⋯*A*	*D*—H⋯*A*
C6—H6⋯O1*W*	0.987 (19)	2.42 (2)	3.340 (2)	155 (2)
N11—H11⋯O1*W*	0.92 (2)	1.94 (2)	2.845 (2)	167 (2)
C19—H19⋯O1*W*	0.977 (19)	2.49 (2)	3.439 (2)	164 (1)
C8—H8⋯O20^i^	1.02 (2)	2.45 (2)	3.427 (2)	160 (2)
O1*W*—H1*W*1⋯N1^ii^	0.91 (2)	1.93 (2)	2.807 (2)	161 (2)
C21—H21*C*⋯CgA^iii^	0.94 (2)	2.83 (2)	3.550 (2)	135 (2)
C21—H21*B*⋯CgB^iv^	0.97 (2)	2.72 (2)	3.503 (2)	138 (2)
O1*W*—H1*W*2⋯CgD^iii^	0.82 (3)	2.62 (3)	3.310 (2)	143 (2)

Symmetry codes: (i) 
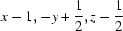
; (ii) 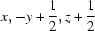; (iii) 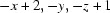; (iv) 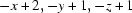.

**Fig. 1.**
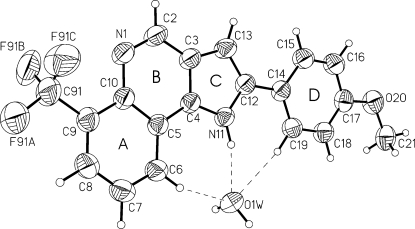
Anisotropic ellipsoid representation of the compound 1 . H2O showing the atom labelling scheme. Ellipsoids are drawn at 50% probability level, hydrogen atoms are depicted as spheres with arbitrary radii. Hydrogen bonds and weaker C—H···O contacts (cf. Comment Section) are shown as dashed lines.

**Fig. 2.**
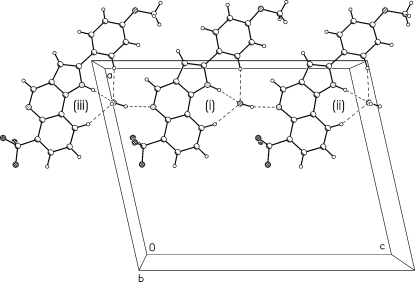
Hydrogen bonded chain along [001]; N—H···O, O—H···N, and C—H···O hydrogen bonds are shown as dashed lines.

**Fig. 3.**
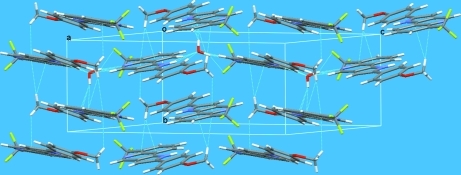
The crystal packing; hydrogen bonds and O—H···π and C—H···π contacts are shown as dashed lines.

## supplementary crystallographic information

### Comment

There is only one crystal structure of a compound having a pyrrolo[3,2-*c*]quinoline skeleton in the Cambridge Crystallographic Database (Allen, 2002; Version 5.31 of November 2009, last update Feb. 2010): 1-phenyl-2,3,4-tris(trifluoromethyl)pyrrolo[2,3-*c*]quinoline (Fan & Chen, 1987). The only other similar structurally characterized compounds are derivatives of 1*H*-pyrrolo[2,3-*h*]quinoline, namely 2-(4-pyridyl)-pyrrolo[3,2-*h*]quinoline (Lynch *et al.*, 2001) and 2-phenylpyrrolo[2,3-*h*]quinoline dihydrate (Lynch & McClenaghan, 2002). Here we present the results of the crystal structure determination of 2-(4-methoxyphenyl)-6-(trifluoromethyl)-1*H*-pyrrolo[3,2-*c*]quinoline hydrate(1 . H_2_O, Scheme 1).

Two planar systems in (1), pyrroloquinoline (planar within 0.0171 (4) Å) and the phenyl ring (0.0050 (11) Å) make a dihedral angle of 10.94 (4)°. Therefore, the complete molecule (without F atoms) is approximately planar. The methoxy group also is not twisted significantly (5.4 (1)°) with respect to the phenyl ring plane. Bond angles within the phenyl ring are influenced by the presence of substituents. As expected for *p*-disubstitution, the influences are almost additive. The sum of values given by Domenicano (1988) or found in the CSD for mono-substituted phenyl rings are very close to the actual values in (1).

The primary motif of the crystal packing is a chain of alternate water and 1 molecules (Fig. 2, Table 1). In that chain (C^2^_2_(8) using graph set notation: Bernstein *et al.*, 1995) both components act as hydrogen bond donor and acceptor. N11—H11 group of 1 donates hydrogen for the N—H···O1W (water) hydrogen bond, and the water molecule acts as a donor for the *O*—*H*···N1(quinoline) hydrogen bond. Due to the steric requirement, the O1W oxygen atom is also in close contact with the adjacent H6 and H19 hydrogen atoms. Because of the geometric parameters of these interactions (Table 1), they might be regarded as the secondary, weak hydrogen bonds. The remaining hydrogen atom of the water molecule does not take part in "classical" hydrogen bonds; instead this O—H bond points toward the phenyl ring of the neighbouring molecule, probably making the O—H(water)···π weak hydrogen bond. Such hydrogen bonds were described by Atwood *et al.* (1991), and they are supposed to play a role in the biological systems. There are some 300 cases of such short contacts in the CSD. In 1, O—H···π hydrogen bonds together with another weak interactions of C—H···O, C—H···π and π···π type, connect the neighbouring chains (Table 1, Fig. 3).

### Experimental

1-(4-methoxyphenyl)ethanone [8-(trifluoromethyl)quinolin-4-yl]hydrazone (1.8 g, 5 mmoles), prepared according to the previously described method (Dutkiewicz *et al.*, 2010) was added to 5 ml of diphenyl ether and the mixture was heated at 523 K in an oil bath for 6 hrs. After cooling, ether was added until the solution became cloudy. Further cooling resulted in precipitation of the product (I), which was collected by filtration. Recrystallization was performed at room temperature from ethanol, m.p.: 401-403 K. Analysis found: C 66.56, H 3.79, N 8.23%; C_19_H_13_F_3_N_2_O, requires: C 66.66, H 3.83, N 8.18%

### Refinement

Hydrogen atoms were located in the difference Fourier maps and isotropically refined.

### Crystal data

**Table:**

C_19_H_13_F_3_N_2_O·H_2_O	*F*(000) = 744
*M**_r_* = 360.33	*D*_x_ = 1.418 Mg m^−^^3^
Monoclinic, *P*2_1_/*c*	Cu *K*α radiation, λ = 1.54178 Å
Hall symbol: -P 2ybc	Cell parameters from 3587 reflections
*a* = 13.838 (1) Å	θ = 3.3–75.2°
*b* = 7.0432 (5) Å	µ = 0.99 mm^−^^1^
*c* = 17.758 (2) Å	*T* = 295 K
β = 102.743 (8)°	Plate, colourless
*V* = 1688.2 (2) Å^3^	0.4 × 0.2 × 0.1 mm
*Z* = 4	

### Data collection

**Table:**

Oxford Diffraction SuperNova (single source at offset) Atlas diffractometer	3304 independent reflections
Radiation source: SuperNova (Cu) X-ray Source	2767 reflections with *I* > 2σ(*I*)
mirror	*R*_int_ = 0.013
Detector resolution: 5.2679 pixels mm^-1^	θ_max_ = 75.3°, θ_min_ = 3.3°
ω scans	*h* = −17→15
Absorption correction: multi-scan (*CrysAlis PRO*; Oxford Diffraction, 2009)	*k* = −8→5
*T*_min_ = 0.340, *T*_max_ = 1.000	*l* = −22→19
5601 measured reflections	

### Refinement

**Table:**

Refinement on *F*^2^	Secondary atom site location: difference Fourier map
Least-squares matrix: full	Hydrogen site location: inferred from neighbouring sites
*R*[*F*^2^ > 2σ(*F*^2^)] = 0.041	All H-atom parameters refined
*wR*(*F*^2^) = 0.128	*w* = 1/[σ^2^(*F*_o_^2^) + (0.0763*P*)^2^ + 0.1838*P*] where *P* = (*F*_o_^2^ + 2*F*_c_^2^)/3
*S* = 1.07	(Δ/σ)_max_ = 0.009
3304 reflections	Δρ_max_ = 0.19 e Å^−^^3^
296 parameters	Δρ_min_ = −0.21 e Å^−^^3^
0 restraints	Extinction correction: *SHELXL97* (Sheldrick, 2008), Fc^*^=kFc[1+0.001xFc^2^λ^3^/sin(2θ)]^-1/4^
Primary atom site location: structure-invariant direct methods	Extinction coefficient: 0.0039 (10)

### Special details

**Table:**

Geometry. All s.u.'s (except the s.u. in the dihedral angle between two l.s. planes) are estimated using the full covariance matrix. The cell s.u.'s are taken into account individually in the estimation of s.u.'s in distances, angles and torsion angles; correlations between s.u.'s in cell parameters are only used when they are defined by crystal symmetry. An approximate (isotropic) treatment of cell s.u.'s is used for estimating s.u.'s involving l.s. planes.
Refinement. Refinement of *F*^2^ against ALL reflections. The weighted *R*-factor wR and goodness of fit *S* are based on *F*^2^, conventional *R*-factors *R* are based on *F*, with *F* set to zero for negative *F*^2^. The threshold expression of *F*^2^ > σ(*F*^2^) is used only for calculating *R*-factors(gt) etc. and is not relevant to the choice of reflections for refinement. *R*-factors based on *F*^2^ are statistically about twice as large as those based on *F*, and *R*- factors based on ALL data will be even larger.

### Fractional atomic coordinates and isotropic or equivalent isotropic displacement parameters (Å^2^)

**Table:**

	*x*	*y*	*z*	*U*_iso_*/*U*_eq_	
N1	0.78945 (9)	0.29250 (19)	0.16925 (7)	0.0550 (3)	
C2	0.88353 (11)	0.3157 (3)	0.20243 (8)	0.0585 (4)	
H2	0.9306 (14)	0.344 (3)	0.1687 (10)	0.071 (5)*	
C3	0.92113 (10)	0.3055 (2)	0.28263 (8)	0.0510 (3)	
C4	0.85387 (10)	0.26693 (18)	0.32912 (7)	0.0439 (3)	
C5	0.75150 (10)	0.24194 (18)	0.29686 (8)	0.0457 (3)	
C6	0.67970 (11)	0.2042 (2)	0.34033 (9)	0.0564 (4)	
H6	0.6994 (13)	0.196 (3)	0.3971 (11)	0.069 (5)*	
C7	0.58263 (12)	0.1830 (3)	0.30423 (10)	0.0684 (5)	
H7	0.5303 (15)	0.158 (3)	0.3354 (11)	0.080 (6)*	
C8	0.55295 (12)	0.1992 (3)	0.22380 (10)	0.0668 (4)	
H8	0.4799 (16)	0.183 (3)	0.1986 (11)	0.080 (6)*	
C9	0.62098 (11)	0.2358 (2)	0.18026 (9)	0.0564 (4)	
C91	0.58623 (13)	0.2559 (3)	0.09412 (11)	0.0790 (6)	
F91A	0.48893 (9)	0.2321 (3)	0.07127 (7)	0.1234 (6)	
F91B	0.62834 (10)	0.1319 (2)	0.05506 (7)	0.1118 (5)	
F91C	0.60642 (9)	0.4282 (2)	0.06897 (7)	0.1051 (5)	
C10	0.72299 (10)	0.25735 (19)	0.21521 (8)	0.0474 (3)	
N11	0.90552 (8)	0.26236 (16)	0.40374 (6)	0.0451 (3)	
H11	0.8790 (14)	0.233 (2)	0.4455 (11)	0.065 (5)*	
C12	1.00454 (10)	0.2982 (2)	0.40618 (8)	0.0472 (3)	
C13	1.01616 (10)	0.3251 (2)	0.33251 (8)	0.0560 (4)	
H13	1.0773 (14)	0.354 (3)	0.3175 (10)	0.073 (5)*	
C14	1.07851 (10)	0.30124 (19)	0.47970 (8)	0.0469 (3)	
C15	1.17510 (10)	0.3653 (2)	0.48185 (9)	0.0557 (4)	
H15	1.1925 (12)	0.408 (3)	0.4342 (10)	0.067 (5)*	
C16	1.24628 (11)	0.3636 (2)	0.54972 (9)	0.0590 (4)	
H16	1.3115 (14)	0.409 (3)	0.5519 (10)	0.072 (5)*	
C17	1.22347 (10)	0.3006 (2)	0.61766 (8)	0.0528 (3)	
C18	1.12861 (11)	0.2388 (2)	0.61730 (9)	0.0547 (4)	
H18	1.1117 (13)	0.195 (2)	0.6656 (11)	0.064 (5)*	
C19	1.05731 (10)	0.2390 (2)	0.54829 (8)	0.0525 (3)	
H19	0.9913 (14)	0.193 (2)	0.5499 (10)	0.064 (5)*	
O20	1.29904 (8)	0.30707 (18)	0.68177 (6)	0.0660 (3)	
C21	1.27720 (15)	0.2604 (3)	0.75398 (10)	0.0669 (5)	
H21A	1.3371 (18)	0.280 (3)	0.7903 (13)	0.090 (7)*	
H21B	1.2269 (17)	0.345 (3)	0.7653 (12)	0.089 (6)*	
H21C	1.2575 (14)	0.133 (3)	0.7546 (11)	0.078 (6)*	
O1W	0.81044 (9)	0.12279 (19)	0.51922 (6)	0.0610 (3)	
H1W1	0.7916 (17)	0.165 (3)	0.5623 (13)	0.092 (7)*	
H1W2	0.802 (2)	0.008 (4)	0.5170 (15)	0.121 (10)*	

### Atomic displacement parameters (Å^2^)

**Table:**

	*U*^11^	*U*^22^	*U*^33^	*U*^12^	*U*^13^	*U*^23^
N1	0.0483 (6)	0.0776 (8)	0.0393 (6)	0.0045 (6)	0.0100 (5)	−0.0023 (5)
C2	0.0472 (7)	0.0893 (11)	0.0408 (7)	0.0035 (7)	0.0132 (6)	0.0002 (7)
C3	0.0445 (7)	0.0674 (8)	0.0416 (7)	0.0037 (6)	0.0105 (5)	−0.0016 (6)
C4	0.0430 (7)	0.0504 (7)	0.0378 (6)	0.0029 (5)	0.0077 (5)	−0.0014 (5)
C5	0.0448 (7)	0.0500 (7)	0.0414 (7)	0.0012 (5)	0.0076 (5)	−0.0008 (5)
C6	0.0491 (8)	0.0735 (9)	0.0463 (7)	−0.0054 (7)	0.0100 (6)	0.0035 (7)
C7	0.0489 (8)	0.0975 (12)	0.0597 (9)	−0.0095 (8)	0.0143 (7)	0.0046 (9)
C8	0.0443 (8)	0.0930 (12)	0.0600 (9)	−0.0049 (8)	0.0046 (7)	−0.0004 (8)
C9	0.0463 (8)	0.0711 (9)	0.0487 (8)	0.0016 (6)	0.0043 (6)	−0.0033 (6)
C91	0.0496 (9)	0.1272 (17)	0.0557 (10)	−0.0007 (10)	0.0016 (7)	−0.0078 (10)
F91A	0.0517 (6)	0.2442 (19)	0.0643 (7)	−0.0149 (8)	−0.0089 (5)	0.0008 (8)
F91B	0.0891 (8)	0.1757 (14)	0.0664 (6)	−0.0034 (8)	0.0080 (6)	−0.0496 (8)
F91C	0.0881 (8)	0.1500 (12)	0.0710 (7)	0.0129 (8)	0.0040 (6)	0.0398 (8)
C10	0.0460 (7)	0.0539 (7)	0.0415 (7)	0.0029 (5)	0.0080 (6)	−0.0027 (5)
N11	0.0421 (6)	0.0557 (6)	0.0369 (5)	0.0007 (4)	0.0074 (4)	0.0000 (4)
C12	0.0403 (6)	0.0557 (7)	0.0446 (7)	0.0039 (5)	0.0068 (5)	−0.0019 (5)
C13	0.0410 (7)	0.0829 (10)	0.0446 (7)	0.0022 (7)	0.0108 (6)	0.0002 (7)
C14	0.0412 (7)	0.0537 (7)	0.0444 (7)	0.0042 (5)	0.0061 (5)	−0.0023 (5)
C15	0.0445 (7)	0.0705 (9)	0.0514 (8)	−0.0003 (6)	0.0090 (6)	0.0010 (7)
C16	0.0412 (7)	0.0743 (10)	0.0596 (8)	−0.0019 (7)	0.0069 (6)	−0.0005 (7)
C17	0.0430 (7)	0.0580 (8)	0.0515 (7)	0.0063 (6)	−0.0022 (6)	−0.0032 (6)
C18	0.0495 (8)	0.0667 (9)	0.0457 (8)	0.0007 (6)	0.0054 (6)	0.0027 (6)
C19	0.0415 (7)	0.0682 (9)	0.0459 (7)	−0.0012 (6)	0.0057 (6)	−0.0004 (6)
O20	0.0488 (6)	0.0855 (8)	0.0553 (6)	0.0016 (5)	−0.0065 (5)	−0.0003 (5)
C21	0.0688 (11)	0.0694 (11)	0.0532 (9)	0.0023 (8)	−0.0063 (8)	−0.0012 (8)
O1W	0.0696 (7)	0.0718 (8)	0.0449 (5)	−0.0025 (6)	0.0199 (5)	0.0002 (5)

### Geometric parameters (Å, °)

**Table:**

N1—C2	1.3155 (19)	N11—H11	0.92 (2)
N1—C10	1.3800 (18)	C12—C13	1.3662 (19)
C2—C3	1.4061 (19)	C12—C14	1.4707 (18)
C2—H2	1.00 (2)	C13—H13	0.96 (2)
C3—C4	1.4004 (19)	C14—C19	1.385 (2)
C3—C13	1.4210 (19)	C14—C15	1.403 (2)
C4—N11	1.3601 (17)	C15—C16	1.378 (2)
C4—C5	1.4170 (19)	C15—H15	0.977 (18)
C5—C6	1.411 (2)	C16—C17	1.386 (2)
C5—C10	1.4202 (19)	C16—H16	0.950 (19)
C6—C7	1.363 (2)	C17—O20	1.3663 (16)
C6—H6	0.987 (19)	C17—C18	1.382 (2)
C7—C8	1.401 (2)	C18—C19	1.394 (2)
C7—H7	1.02 (2)	C18—H18	0.988 (19)
C8—C9	1.368 (2)	C19—H19	0.977 (19)
C8—H8	1.02 (2)	O20—C21	1.419 (2)
C9—C10	1.419 (2)	C21—H21A	0.94 (2)
C9—C91	1.506 (2)	C21—H21B	0.97 (2)
C91—F91B	1.327 (2)	C21—H21C	0.94 (2)
C91—F91A	1.328 (2)	O1W—H1W1	0.91 (2)
C91—F91C	1.344 (3)	O1W—H1W2	0.82 (3)
N11—C12	1.3845 (17)		
			
C2—N1—C10	118.69 (12)	C4—N11—H11	124.9 (11)
N1—C2—C3	123.77 (13)	C12—N11—H11	125.7 (11)
N1—C2—H2	117.9 (10)	C13—C12—N11	108.67 (12)
C3—C2—H2	118.3 (10)	C13—C12—C14	129.95 (13)
C4—C3—C2	117.49 (13)	N11—C12—C14	121.38 (12)
C4—C3—C13	107.18 (12)	C12—C13—C3	107.17 (12)
C2—C3—C13	135.34 (14)	C12—C13—H13	126.2 (11)
N11—C4—C3	107.71 (12)	C3—C13—H13	126.6 (11)
N11—C4—C5	130.89 (12)	C19—C14—C15	117.58 (13)
C3—C4—C5	121.39 (12)	C19—C14—C12	122.34 (12)
C6—C5—C4	124.31 (13)	C15—C14—C12	120.07 (13)
C6—C5—C10	120.15 (13)	C16—C15—C14	120.92 (14)
C4—C5—C10	115.54 (12)	C16—C15—H15	119.7 (10)
C7—C6—C5	120.19 (14)	C14—C15—H15	119.4 (10)
C7—C6—H6	119.7 (11)	C15—C16—C17	120.54 (14)
C5—C6—H6	120.1 (11)	C15—C16—H16	121.5 (11)
C6—C7—C8	120.51 (15)	C17—C16—H16	117.9 (11)
C6—C7—H7	120.5 (11)	O20—C17—C18	124.48 (14)
C8—C7—H7	119.0 (11)	O20—C17—C16	115.81 (13)
C9—C8—C7	120.52 (15)	C18—C17—C16	119.70 (13)
C9—C8—H8	120.9 (12)	C17—C18—C19	119.43 (14)
C7—C8—H8	118.6 (12)	C17—C18—H18	120.1 (11)
C8—C9—C10	121.00 (14)	C19—C18—H18	120.5 (11)
C8—C9—C91	119.11 (14)	C14—C19—C18	121.83 (14)
C10—C9—C91	119.88 (14)	C14—C19—H19	120.8 (10)
F91B—C91—F91A	106.84 (17)	C18—C19—H19	117.4 (10)
F91B—C91—F91C	105.85 (17)	C17—O20—C21	117.95 (13)
F91A—C91—F91C	106.47 (18)	O20—C21—H21A	104.7 (14)
F91B—C91—C9	112.99 (17)	O20—C21—H21B	110.5 (13)
F91A—C91—C9	111.93 (16)	H21A—C21—H21B	109.4 (17)
F91C—C91—C9	112.29 (16)	O20—C21—H21C	110.6 (12)
N1—C10—C5	123.12 (13)	H21A—C21—H21C	110.3 (17)
N1—C10—C9	119.26 (13)	H21B—C21—H21C	111.1 (18)
C5—C10—C9	117.63 (13)	H1W1—O1W—H1W2	107 (2)
C4—N11—C12	109.27 (11)		
			
C10—N1—C2—C3	0.5 (3)	C8—C9—C10—N1	179.42 (16)
N1—C2—C3—C4	0.5 (3)	C91—C9—C10—N1	−1.4 (2)
N1—C2—C3—C13	−179.65 (17)	C8—C9—C10—C5	−0.8 (2)
C2—C3—C4—N11	179.62 (13)	C91—C9—C10—C5	178.39 (15)
C13—C3—C4—N11	−0.26 (16)	C3—C4—N11—C12	0.29 (15)
C2—C3—C4—C5	−1.0 (2)	C5—C4—N11—C12	−179.04 (13)
C13—C3—C4—C5	179.15 (13)	C4—N11—C12—C13	−0.21 (16)
N11—C4—C5—C6	−0.2 (2)	C4—N11—C12—C14	−179.70 (12)
C3—C4—C5—C6	−179.49 (14)	N11—C12—C13—C3	0.05 (17)
N11—C4—C5—C10	179.70 (13)	C14—C12—C13—C3	179.47 (14)
C3—C4—C5—C10	0.45 (19)	C4—C3—C13—C12	0.12 (17)
C4—C5—C6—C7	179.73 (15)	C2—C3—C13—C12	−179.72 (18)
C10—C5—C6—C7	−0.2 (2)	C13—C12—C14—C19	−168.59 (16)
C5—C6—C7—C8	−0.3 (3)	N11—C12—C14—C19	10.8 (2)
C6—C7—C8—C9	0.2 (3)	C13—C12—C14—C15	10.1 (2)
C7—C8—C9—C10	0.3 (3)	N11—C12—C14—C15	−170.52 (13)
C7—C8—C9—C91	−178.87 (18)	C19—C14—C15—C16	0.8 (2)
C8—C9—C91—F91B	−121.27 (19)	C12—C14—C15—C16	−177.96 (14)
C10—C9—C91—F91B	59.5 (2)	C14—C15—C16—C17	−0.8 (2)
C8—C9—C91—F91A	−0.6 (3)	C15—C16—C17—O20	−179.21 (14)
C10—C9—C91—F91A	−179.81 (17)	C15—C16—C17—C18	0.0 (2)
C8—C9—C91—F91C	119.09 (18)	O20—C17—C18—C19	179.80 (14)
C10—C9—C91—F91C	−60.1 (2)	C16—C17—C18—C19	0.6 (2)
C2—N1—C10—C5	−1.0 (2)	C15—C14—C19—C18	−0.1 (2)
C2—N1—C10—C9	178.74 (15)	C12—C14—C19—C18	178.60 (13)
C6—C5—C10—N1	−179.48 (14)	C17—C18—C19—C14	−0.6 (2)
C4—C5—C10—N1	0.6 (2)	C18—C17—O20—C21	−4.8 (2)
C6—C5—C10—C9	0.7 (2)	C16—C17—O20—C21	174.42 (15)
C4—C5—C10—C9	−179.21 (12)		

### Hydrogen-bond geometry (Å, °)

**Table:**

*CgA*, *CgB*, *CgD* are the centroids of the C5–C9,C1C, N1,C2–C5,C1C and C14–C19 rings, respectively

**Table:**

*D*—H···*A*	*D*—H	H···*A*	*D*···*A*	*D*—H···*A*
C6—H6···O1W	0.987 (19)	2.42 (2)	3.340 (2)	155 (2)
N11—H11···O1W	0.92 (2)	1.94 (2)	2.845 (2)	167 (2)
C19—H19···O1W	0.977 (19)	2.49 (2)	3.439 (2)	164 (1)
C8—H8···O20^i^	1.02 (2)	2.45 (2)	3.427 (2)	160 (2)
O1W—H1W1···N1^ii^	0.91 (2)	1.93 (2)	2.807 (2)	161 (2)
C21—H21C···CgA^iii^	0.94 (2)	2.83 (2)	3.550 (2)	135 (2)
C21—H21B···CgB^iv^	0.97 (2)	2.72 (2)	3.503 (2)	138 (2)
O1W—H1W2···CgD^iii^	0.82 (3)	2.62 (3)	3.310 (2)	143 (2)

Symmetry codes: (i) *x*−1, −*y*+1/2, *z*−1/2; (ii) *x*, −*y*+1/2, *z*+1/2; (iii) −*x*+2, −*y*, −*z*+1; (iv) −*x*+2, −*y*+1, −*z*+1.

## References

[bb1] Allen, F. H. (2002). *Acta Cryst.* B**58**, 380–388. 10.1107/s0108768102003890 12037359

[bb2] Altomare, A., Cascarano, G., Giacovazzo, C. & Guagliardi, A. (1993). *J. Appl. Cryst* **26**, 343-350.

[bb3] Atwood, J. L., Hamada, F., Robinson, K. D., Orr, G. W. & Vincent, R. L. (1991). *Nature (London)*, **349**, 683–684.

[bb4] Bernstein, J., Davis, R. E., Shimoni, L. & Chang, N.-L. (1995). *Angew. Chem. Int. Ed. Engl.* **34**, 1555–1573.

[bb5] Domenicano, A. (1988). *Stereochemical Applications of Gas-Phase Electron Diffraction*, edited by I. Hargittai & M. Hargittai, pp. 281–324. New York: VCH.

[bb6] Dutkiewicz, G., Mayekar, A. N., Yathirajan, H. S., Narayana, B. & Kubicki, M. (2010). *Acta Cryst.* E**66**, o874. 10.1107/S1600536810009475 PMC2984078 21580694

[bb7] Fan, Z. & Chen, L. (1987). *Acta Cryst.* C**43**, 2206–2209.

[bb8] Lynch, D. E. & McClenaghan, I. (2002). *Acta Cryst.* E**58**, o1150–o1151.

[bb9] Lynch, D. E., McClenaghan, I. & Light, M. E. (2001). *Acta Cryst.* E**57**, o56–o57. 10.1107/s0108270101005819 11443256

[bb10] Macrae, C. F., Bruno, I. J., Chisholm, J. A., Edgington, P. R., McCabe, P., Pidcock, E., Rodriguez-Monge, L., Taylor, R., van de Streek, J. & Wood, P. A. (2008). *J. Appl. Cryst.* **41**, 466–470.

[bb11] Oxford Diffraction (2009). *CrysAlis PRO* Oxford Diffraction Ltd, Yarnton, England.

[bb12] Sheldrick, G. M. (2008). *Acta Cryst.* A**64**, 112–122. 10.1107/S0108767307043930 18156677

[bb13] Siemens (1989). *Stereochemical Workstation Operation Manual* Siemens Analytical X-ray Instruments Inc., Madison, Wisconsin, USA.

